# Preventing AVF thrombosis: the rationale and design of the Omega-3 fatty acids (Fish Oils) and Aspirin in Vascular access OUtcomes in REnal Disease (FAVOURED) study

**DOI:** 10.1186/1471-2369-10-1

**Published:** 2009-01-21

**Authors:** Ashley Irish, Gursharan Dogra, Trevor Mori, Elaine Beller, Stephane Heritier, Carmel Hawley, Peter Kerr, Amanda Robertson, Johan Rosman, Peta-Anne Paul-Brent, Melissa Starfield, Kevan Polkinghorne, Alan Cass

**Affiliations:** 1 Renal Unit, Royal Perth Hospital, Perth, WA, Australia; 2 Renal Unit, Sir Charles Gairdner Hospital, Perth, WA, Australia; 3 School of Medicine and Pharmacology, University of Western Australia, Perth, WA, Australia; 4 Australian Kidney Trials Network, School of Population Health, University of Queensland, Brisbane, Queensland, Australia; 5 Department of Nephrology, Princess Alexandra Hospital, Brisbane, Queensland, Australia; 6 Department of Nephrology, Monash Medical Centre, Melbourne, VIC, Australia; 7 Department of Surgery, Monash Medical Centre, Melbourne, VIC, Australia; 8 Renal Department North Shore Hospital, Auckland, Auckland, New Zealand; 9 The George Institute for International Health, Sydney, NSW, Australia

## Abstract

**Background:**

Haemodialysis (HD) is critically dependent on the availability of adequate access to the systemic circulation, ideally via a native arteriovenous fistula (AVF). The Primary failure rate of an AVF ranges between 20–54%, due to thrombosis or failure of maturation. There remains limited evidence for the use of anti-platelet agents and uncertainty as to choice of agent(s) for the prevention of AVF thrombosis. We present the study protocol for a randomised, double-blind, placebo-controlled, clinical trial examining whether the use of the anti-platelet agents, aspirin and omega-3 fatty acids, either alone or in combination, will effectively reduce the risk of early thrombosis in de novo AVF.

**Methods/Design:**

The study population is adult patients with stage IV or V chronic kidney disease (CKD) currently on HD or where HD is planned to start within 6 months in whom a planned upper or lower arm AVF is to be the primary HD access. Using a factorial-design trial, patients will be randomised to aspirin or matching placebo, and also to omega-3 fatty acids or matching placebo, resulting in four treatment groups (aspirin placebo/omega-3 fatty acid placebo, aspirin/omega-3 fatty acid placebo, aspirin placebo/omega-3 fatty acid, aspirin/omega-3 fatty acid). Randomisation will be achieved using a dynamic balancing method over the two stratification factors of study site and upper versus lower arm AVF. The medication will be commenced pre-operatively and continued for 3 months post surgery. The primary outcome is patency of the AVF at three months after randomisation. Secondary outcome measures will include functional patency at six and twelve months, primary patency time, secondary (assisted) patency time, and adverse events, particularly bleeding.

**Discussion:**

This multicentre Australian and New Zealand study has been designed to determine whether the outcome of surgery to create de novo AVF can be improved by the use of aspirin and/or omega-3 fatty acids. Recently a placebo-controlled trial has shown that clopidogrel is effective in safely preventing primary AVF thrombosis, but ineffective at increasing functional patency. Our study presents significant differences in the anti-platelet agents used, the study design, and surgical and patient demographics that should contribute further evidence regarding the efficacy of anti-platelet agents.

**Trial Registration:**

Australia & New Zealand Clinical Trials Register (ACTRN12607000569404).

## Background

The incidence and prevalence of severe chronic kidney disease (CKD) is increasing due to ageing of the population and co-morbid conditions such as diabetes. Haemodialysis is the chosen renal replacement therapy for 70% of patients with end-stage kidney disease (ESKD) in Australia. However, effective haemodialysis is critically dependent upon obtaining and maintaining repeated access to the circulation. Delivering the required blood flow (>300 ml/minute) necessary for haemodialysis has long been referred to as the "Achilles' heel" of dialysis. Vascular access options include native arterio-venous fistula (AVF), synthetic arterio-venous graft (AVG) or central venous catheter (CVC). AVF is universally acknowledged as the optimal access option with the best long-term patency, lowest cost and lowest infection rate [1-3] and is the most prevalent access used in Australasia (75%)[4]. Because vascular access related surgical procedures and complications of vascular access represent a high proportion of all admissions in the ESKD population and are a major economic burden for health care providers[5], various international initiatives have made this a focus of quality care improvements[6].

Several studies confirm the risk of all cause death with artificial vascular access devices, compared with AVF as the reference group, is around 1.5 for AVG and 3 for CVC, with similar rates for infectious mortality [2,3,7]. The complications associated with AVG and CVC use are sepsis, especially staphylococcal, vascular malfunction (thrombosis) and death. The increased risk of death is directly attributable to sepsis and the need for interventions to restore patency which in themselves enhance the risk of infection. Furthermore, in comparison with AVF, CVC and AVG have a significantly increased frequency of interventions to maintain patency (assisted patency).

Therefore, while the clinical imperative is to establish AVF in as many patients as possible, primary failure because of early thrombosis and failure of maturation are the major impediments to clinical success[8]. Reports in the literature from the 1970s and 1980s generally describe primary failure rates of between 10 – 20%. A recent meta-analysis examining the primary failure rate of AVF estimated the pooled primary failure rate at 15.3% [9]. However, this meta-analysis included studies back to the 1960s and omitted studies published after the meta-analysis. More contemporary publications are less encouraging with reports of the primary failure rate between 20 – 54%[1]. Consistent with these findings of a high early failure rate, 50% of new patients commence haemodialysis in Australia and New Zealand with a CVC, due to a failed or inadequate AVF[10]. The higher rate of primary failure in the more recent era may reflect changing patient factors – such as older patients and a higher prevalence of diabetes.

Primary failure usually occurs as a result of one of two processes; 1) thrombosis, which usually occurs within weeks of the procedure; 2) inadequate size of the artery or maturation of the vein[8]. Strategies to reduce primary failure rates may therefore include pre-operative identification of unsuitable anatomy by the use of ultrasound, improved surgical technique and pharmacological interventions designed to prevent vessel occlusion (thrombosis).

Early thrombosis is defined as thrombosis within the first 30 days post-operatively[11]. There have been a number of small trials evaluating pharmacological agents aimed at reducing the early thrombosis rate. These trials utilised the anti-platelet agents, aspirin, sulphinpyrazone and ticlopidine and varied in size from as few as 5 patients up to 261 patients. Andrassey et al (n = 92) compared patients given aspirin 500 mg/day for 4 weeks with placebo: the thrombosis rate in the aspirin group was 4% compared with the control group of 24%, OR 0.15 (0.03, 0.73)[12]. Another study [13] with a smaller sample size (n = 68), did not demonstrate a benefit from aspirin. In a study using ticlopidine [14], fistula thrombosis rates for the ticlopidine versus placebo groups were 12% versus 19% (p = 0.10) respectively. Studies using sulphinpyrazone showed variable results, but were underpowered with the largest study enrolling only 36 patients[15]. Pooled data for ticlopidine suggests a reduction in thrombosis rate from 25% to 12% (p < 0.001)[14]. Thus although there is some data to suggest that anti-platelet agents may increase the primary patency of AVF, the limited evidence base and the uncertainty regarding the choice of agent has not supported the widespread use of anti-platelet agents in the prevention of AVF thrombosis. There is a strong clinical need to conduct an adequately powered clinical trial to address the important question of efficacy of anti-platelet therapy in the prevention of early thrombosis of AVF. Since the inception and launch of the FAVOURED study, a US study has been published examining the effect of anti-platelet agent clopidogrel on early AVF thrombosis[16]. We have addressed the findings of this study and their implications for FAVOURED under Discussion below.

Two anti-platelet agents have been chosen to explore their prevention of early thrombosis in AVFs, low dose aspirin and omega-3 fatty acids. Aspirin has been chosen as it has well-established anti-platelet effects, a suggestion of efficacy in the limited studies to date and because it is both inexpensive and an agent familiar to clinicians. Its use as an anti-platelet agent is well established in clinical practice for other purposes relating to thrombosis prevention particularly in patients with established cardiovascular disease. Aspirin's anti-platelet effect is mediated by the inhibition the platelet enzyme cyclo-oxygenase (COX) resulting in blockade of the synthesis of the pro-aggregatory vasoconstrictor, thromboxane A2 (TxA2).

Omega-3 fatty acids have a number of biological effects, which make them an attractive agent for the prevention of vascular access thrombosis. These include the inhibition of platelet aggregation, anti-inflammatory effects and anti-proliferative actions. Omega-3 fatty acids and aspirin both affect the balance between the pro-aggregatory and vasoconstrictor effects of TxA2 and the anti-aggregatory and vasodilator effects of prostacyclin (PGI2) but the mechanism of their effect is different. Omega-3 fatty acids have a weaker inhibitory effect on TxA2 level, their effect mediated by reducing the availability of arachidonic acid (AA), a precursor of TxA2. In addition, omega-3 fatty acids leads to an increase in PGI3 formation (anti-aggregatory and vasodilatation effects equipotent to PGI2)[17]. The anti-inflammatory effect of omega-3 fatty acids is mediated via a reduction in leukotriene and cytokine production. Theoretically, a combination of aspirin and omega-3 fatty acids should result in a more favourable effect on platelet aggregation than either agent used alone.

One study has examined the effects of low-dose aspirin in combination with omega-3 fatty acids on whole blood eicosanoid production[17]. This study demonstrated an *additive *effect of the combination on TxA2 (40% with aspirin alone vs. 62% with the combination) and a smaller reduction in the concentrations of PGI2 and PGI3 compared with aspirin alone. There has only been one study exploring the efficacy of omega-3 fatty acids in the prevention of vascular access thrombosis. This study involved the use of omega-3 fatty acids as a single agent and only explored its use in AVG, not AVF. Importantly, the study demonstrated a substantial reduction in thrombosis at 1 year in patients with AVG[18]. There are other effects of omega-3 fatty acids, which may be particularly beneficial in patients with CKD. Omega-3 fatty acids has been shown to result in an improvement in lipid profile [18-20], to reduce BP and heart rate[21], and to attenuate inflammatory responses [22] and oxidative stress[23]. Other postulated but as yet unproven benefits in the CKD population include a reduction in inflammatory response, cardiovascular mortality and a reduction in uraemic pruritis[24].

The relationship between aspirin use and bleeding is a potential concern for clinicians in patients with CKD. Even in the general population, the use of a chronic low dose of aspirin doubles the risk of serious gastrointestinal bleeding [25] and the theoretical risk may be higher in patients with CKD because of the presence of uraemic induced impairment of haemostasis. There have been published studies[26,27], showing a significant elevation in bleeding times in patients on haemodialysis treatments administered a single dose of aspirin, but surprisingly little evidence-based clinical trial data in this population. This has more recently been explored in the UK-HARP-I study. In this factorial design study involving simvastatin and aspirin as active therapies, allocation to the aspirin group was not associated with an excess of major bleeds (2% in patients on aspirin vs. 3% in patients not on aspirin), but there was a three-fold increase in minor bleeding[28]. Importantly though, in this study, patients received aspirin for 12 months, whereas our study has a much shorter period on therapy of 3 months. In addition, patients on dialysis undergoing renal transplantation have successfully received pre-operative aspirin for the prevention of graft thrombosis without an increased risk of major bleeding [29,30].

Omega-3 fatty acids appear to be well tolerated, even in high doses, with gastrointestinal complaints particularly nausea, vomiting, diarrhoea and non-specific discomfort being the most often reported. In studies looking at the effect of omega-3 fatty acids on bleeding time it has not been found to be significantly prolonged [31]. In studies that have been done thus far in the CKD cohort, there have been no clear effects demonstrated in relation to platelet aggregation and bleeding times. There has only been one report of a serious bleeding event in a single patient in an uncontrolled study[32]. Furthermore, it has been reported that administration of omega-3 fatty acids can protect the gastric mucosa against aspirin-induced injury, the postulated mechanism being that omega-3 fatty acids counteracts the effect of aspirin on the decrease in prostacyclin (combined effect of PGI2 and PGI3)[33]. This is supported by clinical data suggesting that omega-3 fatty acids decreases gastric erosions and ulcers caused by aspirin or alcohol[34]. Thus the combination of aspirin and omega-3 fatty acids may be expected to be additive in terms of efficacy in vascular access thrombosis, but to have a lower risk of bleeding complications than the use of aspirin alone.

We have chosen an oral route of administration for both aspirin and omega-3 fatty acids in this study. The choice of 100 mg for aspirin is based on this dose having adequate anti-platelet aggregation properties and is the dose most commonly used in patients with cardiovascular disease. A 100 mg dose is readily available. The dose of omega-3 fatty acids (4 g daily) has been shown to be well tolerated and improve cardiovascular risk factors in patients at increased risk of cardiovascular disease [35-39]. The marine derived omega-3 fatty acids (commonly known as fish oils) capsules chosen (Omacor^©^), are commercially available and provide the highest concentration of omega-3 fatty acids per gram of oil. The oral absorption of both agents is excellent and thus alternative routes of administration are not required.

The design of a factorial study is based on the hypothesis that aspirin and omega-3 fatty acids will both be effective therapies and that the combination of aspirin and omega-3 fatty acids will be additive, but not synergistic. In relation to safety concerns, although both aspirin and omega-3 fatty acids prolong bleeding time, the combination may be expected to be safer than when aspirin is used alone.

## Methods/Design

Ethics approval for the **Omega-3 fatty acids (Fish Oils) **and **A**spirin in **V**ascular access **OU**tcomes in **RE**nal **D**isease (FAVOURED) trial has been obtained from several local Human Research Ethics Committees and will be obtained in all participating centres prior to study initiation and patient enrolment. The study will be performed in accordance with the 2000 Edinburgh, Scotland Revision of the Declaration of Helsinki, the National Health and Medical Research Committee (NHMRC) Statement on Human Experimentation, Joint NHMRC/AVCC Statement and Guidelines on Research Practice, applicable ICH guidelines and the Therapeutic Goods Administration (TGA). The trial has been registered with Australia & New Zealand Clinical Trials Register (ACTRN12607000569404).

### Study Design

**Figure 1:**
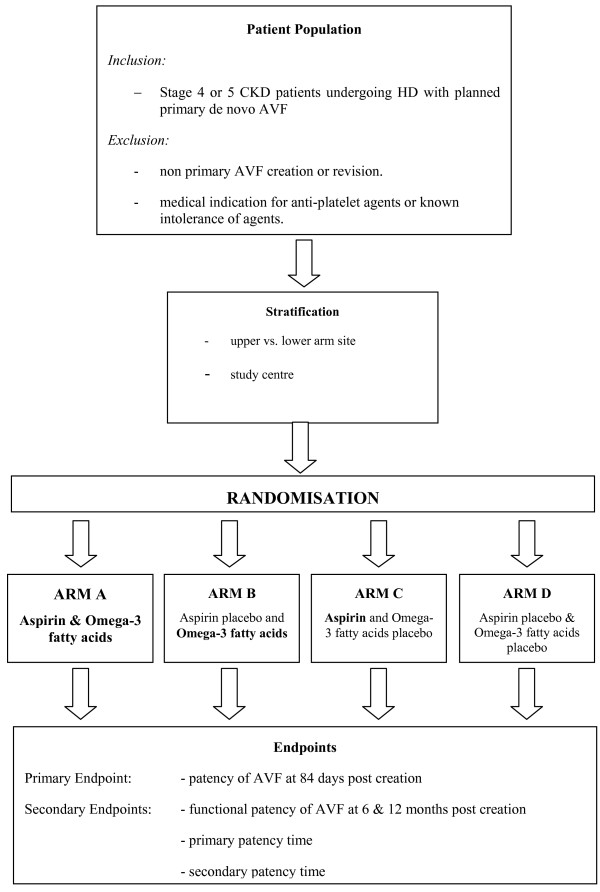
**Schema for the FAVOURED Trial**.

This is a factorial-design trial, where patients are randomised to aspirin or matching placebo, and also to omega-3 fatty acids or matching placebo, resulting in four treatment groups (Figure 1). Randomisation will be achieved using a dynamic balancing method, balancing over the two stratification factors of 1) study site and 2) upper versus lower arm AVF.

Each of the two interventions will be studied using a matching placebo, in order to make this a double-blind trial. At the completion of treatment, patients and investigators will be asked which treatment they believe the patient has been receiving, in order to have a measure of the degree of effectiveness of blinding. Two independent observers will make the primary outcome of patency assessment, unaware of the patient's treatment assignment and treatment course or medical history.

### Patient Population

The study will be multi-centre, and open to all public and private hospital renal units that perform vascular access procedures. The study will be conducted in Australia and New Zealand. In the future international collaboration with sites in Asia and the United Kingdom will be considered.

Patients must meet the following inclusion and exclusion criteria:

#### Inclusion criteria include

The Study population will include adults with the following inclusion criteria:

1. Stage 4 or 5 Chronic Kidney Disease

2. Currently on haemodialysis or haemodialysis is planned to start within 6 months (including patients currently on peritoneal dialysis).

3. Planned AVF will be the primary haemodialysis access mechanism.

4. Surgery to create an arterio-venous fistula in the upper or lower arm is planned.

5. Aged over 19 years

6. Treating team agreeable to patient's involvement in the trial and the patient has given informed consent

#### Exclusion criteria include

1. Revision of existing AVF rather than de novo AVF

2. Medical indication for anti-platelet or thrombolytic agents

3. Known intolerance of agents including hypersensitivity to aspirin, allergy to any other NSAIDs or fish

4. Current use of aspirin within two weeks of commencing trial, or of omega-3 fatty acids within 4 weeks of commencing trial

5. Pregnancy, lactation or intention to fall pregnant during the time course of the study

6. Known bleeding disorder or established diagnosis of active or suspected bleeding

7. History of GI ulcers or bleeding within the last 3 months

8. Platelet count less than 100 × 10^9 ^/L

9. Known active peptic ulcer disease

10. Severe hepatic insufficiency

11. Already receiving anti-coagulation therapy such as warfarin

12. Receiving regular non-steroidal anti-inflammatory (NSAIDS) agents for another indication such as arthritis

13. Syndrome of asthma, rhinitis and nasal polyps if uncontrolled on usual therapy

14. Plan to have other (non-access) surgery within 2 weeks of trial medication period where in the opinion of the investigator aspirin or omega-3 fatty acids would be contraindicated for the planned procedure.

15. Potential non-compliance with treatment regimen in the view of the treating clinicians

16. Involved in another clinical trial where the intervention being trialled is likely to confound the outcome of this trial

17. Previously randomised to this trial.

### Outcome measures

The **primary outcome measure **is (unassisted) patency of the AVF at three months after randomisation. This is defined as the presence of an audible bruit throughout systole and diastole over the site of the arterio-venous anastomosis, without the need for surgical or radiological intervention from the time of creation until the time of assessment. The assessment of patency will be made at this time even if the AVF is not yet used for haemodialysis[11].

#### Secondary outcome measures

1. Functional patency at 6 months. This is defined as the ability to use the AVF for dialysis to the satisfaction of the clinician(s), without the need to use alternative dialysis access, or to delay commencement of dialysis due to access unsuitability.

2. Functional patency at 12 months. This is defined as for the outcome measure functional patency at 6 month but is intended to capture the results of patients whose commencement of dialysis is delayed beyond 6 months. In patients who have not needed to start dialysis, assessment of functional patency will be delayed until commencement of dialysis.

3. Time to failure of primary patency: Time from AVF creation to need for first intervention. An event is defined as revision surgery, need for alternative access, or abandonment of AVF whichever comes first. Censored observations are defined as regaining renal function such that dialysis is not needed or still having a patent AVF at time of study analysis. There will be a minimum of 12 months follow up time for each patient.

4. Time to failure of assisted patency. This is defined as time from AVF creation to abandonment of access. That is, radiological and surgical interventions to preserve or restore patency do not constitute failure. An event is defined as abandonment of the AVF as dialysis access. Censored observations will be as for time to failure of primary patency above.

5. Adverse events. All serious adverse events will be collected. The analysis of this secondary outcome will focus particularly on bleeding events.

**Exploratory outcome measures **are also defined, in order to confirm the validity of the primary outcome measure, and to provide a means of comparison with other previous trials:

1. Patency in the first 24 hours post surgery: determined by the presence or absence of a bruit over the site of the anastomosis, with standard stethoscope.

2. Patency at 6 weeks post surgery: determined by the presence or absence of a bruit over the site of the anastomosis.

3. Time to first cannulation of the AVF: time taken as time of surgery until the first attempt at access cannulation.

4. Time to consistent cannulation of the AVF: time taken as the time from surgery until that of the 3rd consecutive dialysis session with successful cannulation of the access.

### Study Procedures

Participants will be recruited from public and private hospital renal units providing haemodialysis and vascular access services in Australia and New Zealand. Patients will meet the inclusion and exclusion criteria and be scheduled to undergo surgery for creation of a de novo AV fistula. Processes to identify and screen all potential recruits will be established within each centre with consultation with the Trial Management Committee.

Patient consent forms will be approved by the Human Research Ethics Committee at each participating site prior to the beginning of the trial. A sample consent form and patient information sheet is provided to participating sites. Participating sites will file a copy of the approved consent form and information sheet for their site with the coordinating office. After discussing the trial, ample time will be given to the participant, accompanying person or legal representative to inquire about the trial and decide whether to participate. No person involved with the trial will coerce or unduly influence the decision of a patient to participate in a trial. A copy of the signed consent form and the patient information sheet will be supplied to the participant. Patient consent must be obtained prior to the registration or initiation of trial procedures. Patients will not be randomised until a signed consent form is filed at site and notification is received by the coordinating office.

The Trial Management Committee will monitor the medical literature, and any other relevant information impacting on the continuation of the trial. Consent forms and patient information sheets will be revised should any relevant and important new information become available.

Registration will occur when subjects come for their surgical consultation prior to the operation to create the AVF. The patient will have an initial consultation with a study renal physician or surgeon to discuss study participation. This will include a preliminary eligibility check. Consenting patients will be registered by emailing or faxing a completed registration form to the coordinating centre. The registration form also serves as notification of completion of a patient consent form. The patient will also be randomised at this time. Stratification will occur for study site, incident versus prevalent patient status and upper vs. lower arm site. Patients will be randomised to one of four treatment groups in equal proportion.

Please refer to table 1 for an outline of the timing of the study visits study assessments preformed at those visits.

**Table 1:** Outline of Favoured Study Procedures

Study Visits	Baseline	Randomisation	Surgery	Treatment Period (wks)			Follow-up (mths)					
**Assessment**	**≤ 4 wks prior to surgery**	**≤ 7 Days prior to surgery**		**1**	**6**	**12**	**6**	**12**	**18**	**24**	**30**	**36**

Informed Consent	X											
Study Medication Allocated		X										
Medical History and Physical Exam	X											
Weight & Blood Pressure	X		X	X	X	X	X	X	X	X	X	X
Demographics	X											
Dialysis status	X			X	X	X	X	X	X	X	X	X
Description and status of AVF			X	X	X	X	X	X	X	X	X	X
Concomitant meds	X		X	X	X	X	X					
Adverse events			X	X	X	X	X					
Routine fasting blood & urine samples	X					X						
Batched blood & urine samples	X				X	X						

### Treatment plan and modifications

The study treatment commences on the day prior to the scheduled AVF surgery and will continue for 3 months. Participants will take 100 mg of Aspirin per day p.o. or matching placebo and 4 g of omega-3 fatty acids in divided doses (2 g twice daily) in the form of Omacor capsules, 45% eicosapentaenoic acid (EPA) and 45% docosapentaenoic acid (DHA), or matching placebo.

Compliance will be monitored by capsule/tablet count at scheduled study visits at week 1, 6 and 12. Selected centres will also measure erythrocyte fatty acids for compliance with omega-3 fatty acid intake and urinary thromboxane B2 levels (for patients with urine output) for compliance with aspirin intake. Centres will apply routine biochemistry (lipids, CRP) and haematology analyses as part of patient monitoring.

Study treatment may be discontinued if one of the following occurs:

1. A major bleeding event (e.g. haemorrhagic stroke, gastro-intestinal bleed);

2. The access is abandoned due to thrombosis at any time after the one week assessment;

3. An AVG rather than an AVF is created, or a vein graft is used;

4. Anti platelet therapy or anticoagulation is indicated because of other co morbid events i.e. for example a major cardiovascular event;

5. A female patient becomes pregnant while on study medication. The event should be reported to the trial coordinating centre immediately as a medically important SAE. The event will be reported to Bayer Safety within 24 hours by the coordinating centre and follow up performed through to delivery;

6. The AV anastomosis is created in the lower limbs rather the upper limbs during surgery.

7. Patient receives renal transplant

Study treatment should be continued if:

1. Further access is created (including a new AVF or AVG, peritoneal and venous catheter) because of failure of maturation of the study AVF, i.e. access is still patent;

2. Renal function is recovered; the study AVF can still be measured for patency.

No other anti-platelet or anti-coagulation agent will be permitted during the period the patient is taking study medication.

### Statistical Considerations

#### Sample size calculation for the primary outcome

The event rate for the primary outcome (failure of primary patency) is estimated to be 25% at three months in the control group. If a 30% relative risk reduction is achievable with either aspirin or omega-3 fatty acids, then with 80% power and a significance level of 5%, 1200 study subjects will be required (300 receiving double placebo, 300 active aspirin and placebo omega-3 fatty acids, 300 active omega-3 fatty acids and placebo aspirin, and 300 both active aspirin and active omega-3 fatty acids in a factorial design). This allows for 5% drop-in from placebo to active treatment (either aspirin or omega-3 fatty acids), 5% drop-out from study treatment (to no treatment – assumed equivalent to placebo), and 5% loss to follow-up. This is equivalent to an observed relative risk reduction of 24%. Without adjustment for compliance, the study size needed would have been 932 subjects.

The study is not adequately powered to detect a clinically important difference between the combination of aspirin and omega-3 fatty acids and either treatment alone, but preliminary data on the combination will be obtained. The event rate of the primary outcome, primary failure, is based on review of all papers published in this area. The effect size, a 30% reduction, is based on the smallest size of the effect the proposing clinicians believe would lead to a change in practice (incorporation of intervention) should the trial prove the intervention is efficacious.

#### Sample size calculation for the secondary outcome

Sample size is estimated for the secondary outcome (failure of patency at 6 months) under assumptions of additive or multiplicative effects for each of 3 placebo rates. The calculation below is based on the following assumptions: 5% loss to follow up, 15% drop-in and 5% drop-out. The baseline failure rate of patency at 6 months is (40%, 50% and 60%) in the placebo group, the use of either aspirin or omega-3 fatty acids alone results in a relative risk reduction (RRR) of 25%, i.e. the failure rate becomes respectively 30%, 37.5% and 45% for a patient taking only one drug.

The sample size calculation is based on a marginal analysis (Z-test) with two-sided α = 0.05, power β = 90%. Table 2 presents required sample sizes under additive model (bold) and under multiplicative model

**Table 2:** The sample sizes required under the additive and multiplicative models for the secondary outcome – functional patency

	Total sample size			
	
	Additive model		Multiplicative model	
	
Patency failure rate at 6 months for placebo group	unadjusted	adjusted	unadjusted	adjusted
40%	**874**	**1455**	1156	1812
50%	**620**	**1034**	820	1297
60%	**452**	**752**	596	941

(Unadjusted and adjusted refer to the calculations of sample size without and with adjustment for loss to follow-up (5%), drop-in (15%) and drop-out (5%))

A recently published comparable trial in the United States [16] experienced a placebo rate of 60% for failure of patency at 6 months. We are confident that a sample size based on a placebo rate of at least 50% is a realistic estimate and that the sample of 1,200 recruited for the primary outcome will generally be adequate for the secondary outcome whether the effect is additive or multiplicative.

### Recruitment

Based on data from the ANZDATA renal registry[40], 7,202 patients were receiving dialysis in Australia and New Zealand in December 2004. Importantly, 2042 patients commenced dialysis in Australia and New Zealand in the calendar year 2004. Data obtained from the whole of New Zealand population and access rates for Princess Alexandra Hospital (PAH), Queensland, reveal that 1 procedure is performed per year for every 2.3 – 2.8 patients receiving haemodialysis (total numbers of patients – new patients and prevalent patients). The New Zealand data was obtained by Dr. Mark Marshall (survey from October 1 2000 until September 30, 2001) and by Dr. Carmel Hawley (Queensland) based on prospectively collected data in PAH renal unit from January 2004 until December 2004. In addition further information from the 2005 registry report supports these data. Incident patients require access for dialysis and 27% and 33% of haemodialysis patients in Australia and New Zealand, respectively, were incident patients in 2004. Thus we can expect these patients at the very least to require new vascular access, in addition to existing patients on dialysis who need further access because of failure of previous vascular access procedures. Extrapolation to the whole Australian and New Zealand population, using a conservative estimate of 1 procedure per 3 patients allows us to derive that 2,400 access procedures are performed per year (AVF and AVG). Using a conservative estimate that 75% of access is AVF and not AVG, gives an estimation of 1,800 de-novo AVF creations per year. Assuming we will recruit 22% of these, recruitment should take approximately three years.

This trial is to be conducted by the Australasian Kidney Trials Network (AKTN). The Australian and New Zealand Society of Nephrology, and Kidney Health Australia were instrumental in the development of the AKTN, which is supported by all the major renal units in Australia and New Zealand. The Scientific Committee is representative of the majority of large renal units in Australia and New Zealand. Moreover, the trial has been developed to be as inclusive as possible with an early and relevant primary end-point which should also encourage enrolment. In addition, the Australian and New Zealand renal community has a keen focus on vascular access which has been the subject of a number of recent seminal publications in the area of vascular access. Thus we are confident this trial will be well supported by renal units in Australia and New Zealand.

### Analysis

Analysis of the primary outcome (patency) will be on an intent-to-treat basis. All randomised patients will be analysed in the group they were allocated to, even if they do not receive treatment as allocated, or do not commence treatment. All randomised patients will be included in the analysis.

The primary outcome is measured as the proportion with failure of patency at three months after randomisation. The primary hypothesis will be tested using a chi-square test of proportions to test the null hypotheses that a) the proportion of failures in those allocated to aspirin versus those allocated to placebo aspirin is equal, and b) the proportion of failures in those allocated to omega-3 fatty acids versus those allocated to placebo omega-3 fatty acids is equal. The primary outcome will be measured at 84 days after randomisation, with a permitted tolerance of 7 days prior, and 14 days after the due date of 84 days. Tests of hypotheses will be at the 5% significance level, and 2-sided p-values will be used.

Any patient who does not have a 84-day measure will be counted as failure of patency in the primary analysis. A secondary analysis will be done including only those with a 84-day measure to test the sensitivity of the analysis of the main treatment comparisons to patient exclusions. As this is a short-term intervention, with a relatively short recruitment period, the only planned interim analysis is of safety. This will be conducted at 1/3 and 2/3 of total patient numbers accrued. Similar analyses to the primary one will be undertaken on the secondary outcomes, as well as exploratory analyses looking at the validity of the chosen primary outcome and its correlation with other outcomes, and the prediction of thrombosis from baseline variables such as markers of inflammation.

## Discussion

This multicentre Australian and New Zealand study has been designed to determine whether the outcome of surgery to create AVF can be improved by the use of aspirin and/or omega-3 fatty acids. Vascular access failure is a significant problem for patients receiving dialysis because it increases their risk of infection, hospitalisation and death. The economic costs of the increased hospitalisations arising from inadequate or artificial vascular access are considerable. Effective therapies to prevent failure of primary AVF are therefore warranted. The therapies nominated in the FAVOURED study are widely available and inexpensive.

It is an appropriate time to study the effects of anti-platelet agents in the prevention of AVF thrombosis. The number of patients requiring dialysis is growing and contemporary studies of the natural history of vascular access outcomes show an increase in the incidence of primary failure, which is most likely related to an increasing prevalence of older patients with comorbidities such as diabetes. At the time of inception of the FAVOURED study, there were no adequately powered studies examining the efficacy of anti-platelet agents in the prevention of AVF thrombosis. Subsequent to FAVOURED being launched in Australia and New Zealand, a US group has published a placebo-controlled parallel arm study examining the prevention of early thrombosis in AVFs using Clopidogrel (an antiplatelet agent) with study treatment commencing one day post surgery with a primary outcome of patency at 6 weeks [16]. There was a 12.2% thrombosis rate in the Clopidogrel arm vs. 19.5% in the control arm: RR of 0.63 (0.46–0.97) p = 0.018, adjusted for interim analyses. Suitability of the AVF for dialysis failed in 63% of the clopidogrel group and in 60% of the placebo group (61% overall). There were no differences in adverse events and, surprisingly, bleeding events were very low in both trial arms. The conclusions drawn by the investigators were that although Clopidogrel is effective in safely preventing primary AVF thrombosis, it was ineffective at increasing functional patency and therefore cannot be recommended.

The implications this trial for the FAVOURED Study include the following:

1. The high functional failure rate is especially concerning and much higher than expected. This may reflect aspects related to surgical (e.g. small number of procedures performed by individual surgeons) and nursing care (needling skills for AVF) in the US and/or patients demographics. Furthermore, significant variation between USA and both European and Australian clinical practice could explain the high US study failure and unsuitability rates. In this context, the generalisability of this American study to other countries is uncertain and justifies additional trial confirmation in other countries;

2. Earlier administration of the active agents before surgery (as in FAVOURED) rather than after may lead to an even greater efficacy in relation to the primary thrombosis rate;

3. The rational for trialing two agents in a factorial design remains valid. Omega-3 fatty acids may be of an additional benefit to aspirin by improving vascular endothelial function and smooth muscle relaxation, which may enhance maturation and greater attainment of functional patency;

4. The low bleeding rates and safety data with Clopidogrel are reassuring in the context of uncertainty regarding the safety of anti-platelet therapy in renal failure, given that anecdotally Clopidogrel is considered to be associated with a higher risk of bleeding than aspirin;

5. The greater duration of follow up (minimum of 1 year) in the FAVOURED Study will allow greater ascertainment of the secondary outcome measure with relation to functional patency. This acquires even greater importance in view of the Clopidogrel Study result.

If the trial demonstrates a positive effect of either or both agents, this will support routine use of these agents in clinical practice and lead to a widespread reduction in thrombosis and an increase in functioning AVF. This has significant patient benefits and health system benefits in reduced costs associated with repeat hospitalisations. If the trial demonstrates no effect of the agents, this will suggest that platelet aggregation is not the major mechanism for thrombosis in this population and that efforts to improve AVF patency would then need to be redirected to other mechanisms associated with access failure, such as vascular anatomical function.

## Competing interests

The authors declare that they have no competing interests.

## Authors' contributions

AI: Principal Investigator; conceived study; participated in design and co-ordination; helped to draft manuscript; read and approved the final manuscript. CH: Trial Management Committee member; participated in design and co-ordination; helped to draft manuscript; read and approved the final manuscript. PPB: Trial Management Committee member; participated in design and co-ordination; helped to draft manuscript; read and approved the final manuscript. EB: Trial Management Committee member; participated in design and co-ordination; provided statistical advice; read and approved the final manuscript. SH: Trial Management Committee member; participated in design and co-ordination; provided statistical advice; read and approved the final manuscript. SD: Trial Management Committee member; participated in design and co-ordination; helped to draft manuscript; read and approved the final manuscript. TM: Trial Management Committee member; participated in design and co-ordination; read and approved the final manuscript. JR: Trial Management Committee member; participated in design and co-ordination; read and approved the final manuscript. KP: Trial Management Committee member; participated in design and co-ordination; read and approved the final manuscript. AC: Trial Management Committee member; participated in design and co-ordination; read and approved the final manuscript. PK: Trial Management Committee member; participated in design and co-ordination; read and approved the final manuscript. AR: Trial Management Committee member; participated in design and co-ordination; read and approved the final manuscript. MS: helped to draft manuscript; read and approved the final manuscript.

## Pre-publication history

The pre-publication history for this paper can be accessed here:

http://www.biomedcentral.com/1471-2369/10/1/prepub
